# *Bartonella *seropositivity in children with Henoch-Schonlein purpura

**DOI:** 10.1186/1471-2334-5-21

**Published:** 2005-04-05

**Authors:** Joan L Robinson, Donald W Spady, Errol Prasad, Dorothy McColl, Harvey Artsob

**Affiliations:** 1 Department of Pediatrics and Stollery Children's Hospital, University of Alberta, Edmonton AB, Canada; 2 Dynacare Kasper Medical Laboratories, Edmonton, AB, Canada; 3 Bacterial and Rickettsial Zoonosis Section, National Microbiology Laboratory, Canadian Science Centre for Human and Animal Health, Winnipeg, MB, Canada

## Abstract

**Background:**

An association between Henoch-Schonlein purpura (HSP) and seropositivity for *Bartonella henselae (*BH) has been described. The objective of this study was to see if such an association exists in northern Alberta.

**Methods:**

Immunofluorescent antibody testing utilizing an antigen prepared from *B. henselae *was undertaken on sera from six children with current HSP, 22 children with remote HSP, and 28 controls that were matched for age. Blood from the six children with current HSP was analysed by polymerase chain reaction (PCR) assay with primers derived from the citrate synthase (gltA) gene for the detection of *Bartonella *DNA.

**Results:**

The seropositivity rate for BH was 61% in cases versus 21% in controls (p < 0.03). The PCR assay was negative in all six current cases.

**Conclusion:**

There is an increased seropositivity rate for BH in children with HSP. However, it is not clear if infection with *B. henselae *or a related *Bartonella *species can result in HSP, or if the increased seropositivity is from non-specific or cross-reacting antibodies.

## Background

Henoch Schonlein purpura (HSP) is an idiopathic form of vasculitis, which manifests as a characteristic painless palpable purpuric rash most pronounced on the buttocks and the extensor surfaces of the lower extremities. The vasculitis can also involve the bowel, resulting in abdominal pain. In severe cases, there can be melena, malabsorption, pancreatitis or intussussception [1]. Joint involvement occurs in the majority of cases. Renal involvement occurs in about half of cases, and usually results in a reversible, asymptomatic IgA-mediated nephritis, but about 1% of patients progress to chronic renal failure [1]. Impressive testicular swelling can occur. About 10–20% of patients have recurrences of HSP – typically within a few weeks of the disease appearing to resolve. Evidence of recent infection with group A streptococcus, Epstein-Barr virus (EBV), varicella, parvovirus B19, *Campylobacter*, or *Mycoplasma *have all been found in patients with HSP [2,3], but these organisms do not appear to be etiologic agents.

*Bartonella henselae *is a fastidious gram-negative organism, and is the etiologic agent for cat-scratch disease (CSD) [4]. Less commonly, infection with this organism results in encephalitis, splenic or hepatic abscesses, or osteomyelitis [4]. The organism is presumed to be carried by fleas, which then transmit it to cats, resulting in feline bacteremia. A cat bite or scratch then transmits the organism to humans. A 2002 study from Florida demonstrated that 67% of patients with a recent diagnosis of HSP had serologic evidence of infection with *B. henselae *(versus 14% of a control group) [5]. It is uncertain if this means that *B. henselae *causes HSP or if there is a non-etiologic association between HSP and *B. henselae*.

The objective of this study was to determine if children in northern Alberta with a current or remote diagnosis of HSP have evidence of infection with *B. henselae *or a related *Bartonella *species using both serology and nucleic acid amplification.

## Methods

### Study population

This study was approved by the Health Ethics Review Board of the University of Alberta. Pediatricians were asked to notify us of children with a current or remote diagnosis of HSP, and health records from the Stollery Children's Hospital for 1997–2001 were searched to identify children with this diagnosis. After informed consent was obtained, data were collected from the parents, the patient, and the medical record on the symptoms the child had at the time of diagnosis, the number of recurrences that had occurred to date, the level of exposure to cats, and the results of any biopsies that were done. The diagnosis of HSP was based on either i) the presence of a classic rash with palpable purpuric lesions mainly on lower limbs and buttocks, or ii) an atypical rash and either abdominal pain, joint pain, lower gastrointestinal bleeding, or laboratory evidence of nephritis. Patients were considered to have current HSP if onset of initial or recurrent symptoms was less than 42 days prior to enrollment, recent HSP if symptoms started 42 or more days prior to enrollment but had not yet resolved, and remote HSP if symptoms started 42 or more days prior to enrollment and had resolved.

Paired sera were collected for *B. henselae *serology from test subjects, with the convalescent sera being collected approximately two weeks after the acute sera. Blood was drawn for amplification of *Bartonella*-specific genomic sequences by PCR assay from patients that were considered to have current HSP. *Bartonella henselae *serology was also run on controls that had been matched for age (< 3 yr, 4–7 yr, 8–12 yr, or > 12 yr). Control sera were originally collected for other diagnostic purposes, and no clinical information was available on these children. The technicians were blinded as to the source of the specimens (cases versus controls) and all specimens were run in a single batch.

### Sample size

The assumption was made that if *B. henselae *infection were the sole causative organism of HSP, patients with a current or remote diagnosis of HSP would be sero-positive half the time, as waning antibody titers occur [6]. Assuming that the seropositivity rate in the control group could be as high as 15%, and that the diagnosis of HSP is accurate, enrolling 20 patients would yield the power to demonstrate that over half of cases are seropositive at a confidence interval of 95%.

### Serology

Analysis of sera for immunoglobulin (Ig) G antibodies to *B. henselae *antigen was performed using an indirect immunofluorescence assay (IFA) method with positive and negative controls. A suspension of *B. henselae *(ATCC 49882) in 0.1% formal saline was prepared with bacteria grown in-house on brain heart infusion agar supplemented with 5% sheep blood. The suspension was spotted onto 12-well slides (#ER-202W, Erie Scientific, USA) and air-dried for 1 hour. Slides were fixed in cold acetone for 15 minutes, air-dried, and stored at -80 degrees Celsius. For initial screening, sera from controls and test subjects were diluted 1:32 in FTA Hemagglutination Buffer (#211248, Becton Dickinson, USA). A 1:32 dilution of goat anti-human IgG (whole molecule) FITC conjugated antiserum was used to detect IgG antibodies. Sera reactive at 1:32 were serially titrated two-fold to endpoint. A titer of 1:64 or higher was interpreted as evidence for infection at an undetermined time [6]. A titer of 1:256 or higher was interpreted as evidence for recent infection.

### Polymerase chain reaction

DNA was extracted from six blood clots using a QIAamp DNA Mini Kit (Qiagen, Chatsworth, CA). Polymerase chain reaction amplification to detect *Bartonella*-specific sequences of the citrate synthase gene (gltA) [7] was attempted and found to be negative. Simultaneous amplification with porphobilinogen deaminase gene primers [8] verified that the extracted DNA was of sufficient quality for amplification.

### Statistics

The proportion of seropositivity to *B. henselae *was compared in the cases and controls using a chi-square test. A comparison was done of seropositivity in current cases versus remote cases, and in cases with initial onset of symptoms ≤ 12 months before the serology was drawn, versus those who had onset of symptoms > 12 months earlier. A p value of <0.05 was considered to be significant.

## Results

### Serology

**Figure 1:**
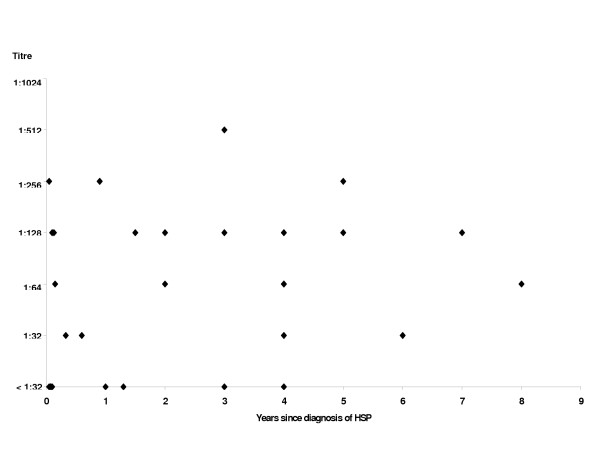
Relationship between results of serology for *B. henselae *and time since onset of Henoch-Schonlein purpura

There were 28 patients (21 males, 7 females) enrolled in the study. In addition to meeting the clinical criteria for HSP, two of these patients had HSP nephritis on renal biopsy, and two others had leukocytoclastic vasculitis on skin biopsy. Six patients had current HSP (five were initial episodes, and one was a recurrence), none had recent HSP, and 22 had remote disease. Seventeen of the 28 cases had contact with cats (nine had contact but no bite or scratch, five had a bite or scratch from an adult cat, and three others had a bite or scratch from a kitten). Results of serology are shown in Table 1. Sixty-one percent of cases were seroreactive to *B. henselae *antigen versus 21% of controls (p < 0.003). Paired sera were drawn 17 and 18 days apart from two patients with current HSP, and had static titers of 1:32 and 1:128, respectively. Figure 1 shows that *B. henselae *titers did not appear to be related to the time that had elapsed since the diagnosis of HSP and Table 2 shows that seropositivity did not seem to be related to the season of the year in which the initial episode of HSP occurred.

**Table 1:** Results of serology for *B. henselae *in patients with Henoch-Schonlein purpura, and in a control group

	n	Titer < 1:64	Titer 1:64 – 1: 128	Titer ≥ 1: 256	Total seropositives
Current HSP	6	3 (50%)	2 (33%)	1 (17%)	3 (50%)
Remote HSP	22	8 (36%)	10 (45%)	4 (18%)	14 (63%)
HSP ≤ 12 months earlier	11	6 (54%)	2 (18%)	3 (27%)	5 (45%)
HSP > 12 months earlier	17	5 (29%)	10 (59%)	2 (12%)	12 (71%)
All HSP	28	11 (39%)	12 (43%)	5 (18%)	17 (61%)
Controls	28	22 (79%)	6 (21%)	0	6 (21%)

**Table 2:** Seasonality of seropositivity for *Bartonella henselae *in patients with Henoch-Schonlein purpura

Month of initial onset of Henoch-Schonlein purpura	Seropositives/Total number tested
January-March	3/8
April-June	1/1
July-September	6/8
October-December	7/11

### Polymerase chain reaction

From each of the six patients diagnosed with current HSP, a single blood sample was collected on one of the following days post onset of symptoms: 7, 18, 19, 20, 24 and 34. All blood samples were negative by PCR assay for *Bartonella*-specific sequences of the citrate synthase gene.

## Discussion

A possible association between HSP and *B. henselae *infection has been noted in one previous study, with an increased seroprevalence in children with recent onset of HSP [5]. In this confirmatory study, there was a significantly increased seroreactivity rate to *B. henselae *antigen in 28 children with a current or remote history of HSP than in controls (p < 0.03). This is the first study to use PCR to attempt to identify *B. henselae *in children with HSP, but the organism was not detected in any of the six cases tested. In the absence of demonstration of genomic sequences specific to *B. henselae*, it is not clear if *B. henselae *causes HSP. Many studies have demonstrated that serological differentiation between *B. henselae *and *B. quintana *infections is impossible, with a cross-reactivity rate between these species of up to 95% [6]. It is possible that the antibodies to *B. henselae *in HSP patients observed here are actually cross-reactive to *B. quintana *or to other bacteria, or are a non-specific reaction to inflammation.

If *B. henselae *or a related *Bartonella *species is the sole causative agent of HSP, one might expect an even higher seroprevalence than the 61% in this study, and 67% in the previous study [5]. Perhaps HSP is a "final common pathway" of infection with multiple organisms including *Bartonella*. Another explanation would be that for the patients with current HSP, serology was run too early in the course of their disease. A study of CSD found that titers peaked between 2 and 16 weeks after onset of symptoms [9]. Serology was drawn 7 to 39 days after the onset of symptoms of HSP in the six patients with current HSP in this study, and mainly within 28 days in the previous study [5]. Obtaining more follow-up titers in patients with HSP might increase the seropositivity rate or demonstrate a rise in titers. Measuring IgM titers to *B. henselae *in patients with acute HSP could also be useful, but potential problems include the lack of sensitivity of the IgM assay, and potential cross-reactivity with IgM to EBV [6].

Another possibility is that the clinical manifestations of HSP constitute an immunologic reaction to an infection that has resolved, such that the titers are already waning when the clinical features of HSP become evident. Therefore, it is possible that some seronegative patients with remote HSP had seroreverted by the time serology was drawn. In the initial study of *B. henselae *serology in CSD, a rapid decline in titers as measured by IFA was noted [6]. A recent study that used an enzyme immunoassay found only 25% of patients maintained IgG to *B. henselae *for more than 12 months [10]. However, in the current study, there was no apparent relationship between the time since diagnosis of HSP and the *B. henselae *titers. Other reasons for false-negative serologic results could be that there are differences in serologic response to different strains of *B. henselae *[6], or that the clinical diagnosis of HSP was incorrect in some cases.

The B. henselae seroprevalence rate in controls of 21% and 14% in the current study and the Florida study [5] respectively are higher than in a summary of North American studies where the rate ranged from 2% [11] to 6% [6]. However, the seroprevalence was 37% in adult blood donors and 18% in children with respiratory illnesses in a study done in British Columbia, Canada [12]. It is well recognized that interpretation of the IFA is subjective, and it is probably not valid to compare titers obtained in different laboratories. Because there have been no previous seroprevalence studies in Alberta, it is not clear if our seroprevalence rate is higher than predicted or if the specificity of the IFA is low. Evidence for the latter is that during the finalization of this manuscript, case and control specimens after being stored for 2 years were re-analyzed by an in-house IFA assay in a different laboratory, and all had titres of <1:50. However, a previous study demonstrated that in-house assay to be less sensitive than a commercial one [13]. A lack of specificity of our IFA would not account for the higher seroprevalence rate in cases than in controls.

*Bartonella *was not detected by PCR in the blood of six acute cases of HSP in the current study, but it is possible that the PCR assay that was used is not sufficiently sensitive, or it may have detected the organisms if done earlier in the course of HSP. In a study of cat-scratch disease, the PCR was positive on the lymph nodes in 10/21 cases, with 9/10 specimens obtained in the first 6 weeks of illness being positive but only 1/11 obtained after 6 weeks of illness being positive [9]. Another explanation for the failure to detect *Bartonella *in the blood could be that the organism is present in the liver, spleen, or lymph nodes in HSP rather than in the blood. If the clinical manifestations of HSP constitute an immunologic reaction to an infection that has resolved, it may be too late to detect the organism by PCR by the time the diagnosis of HSP is evident.

Biopsy of the rash of HSP shows leukoclastic vasculitis. This is a hypersensitivity reaction that can be caused by a wide variety of infections, drugs, insect bites, cold exposure, and malignancies [14]. Although leukoclastic vasculitis has been described in two cases of CSD [5,14], one might expect many more cases if CSD and HSP are caused by the same organism. If *B. henselae *is the cause of HSP, it is a bit surprising that 39 % of our cases had no history of contact with cats. However, in a previous study of serologically proven CSD, only 57% of patients could recall contact with cats [9]. It is possible that humans can acquire infection directly from fleas [4]. In addition, there is some evidence that dogs can transmit *B. henselae *[15].

In summary, patients with a current or remote diagnosis of HSP have increased seropositivity to *B. henselae*. However, no association was found between the antibody titre and the time since the onset of HSP, and blood samples in six patients with acute HSP were negative by PCR assay for *Bartonella*-specific sequences. To prove if *B. henselae *is related to HSP, future studies should perform more extensive serologic follow-up, confirm serologic results with a commercial assay, and perform PCR and cultures on blood and all available tissues as early in the course of the disease as possible. Furthermore, *B. henselae *serology should be done in children with other inflammatory conditions to determine if the seroreactivity is a non-specific inflammatory response.

## Abbreviations

BH – *Bartonella henselae*

CSD – cat-scratch disease

EBV – Epstein Barr virus

HSP – Henoch-Schonlein Purpura

IFA – immunofluorescence assay

PCR – polymerase chain reaction

## Competing interests

The author(s) declare that they have no competing interests.

## Authors' contributions

JR wrote the protocol and the manuscript. DWS did the statistical analysis and reviewed the manuscript. EP, DM, and HA ran the laboratory tests and reviewed the manuscript.

## Pre-publication history

The pre-publication history for this paper can be accessed here:


